# Ten key issues for ecological restoration of territorial space

**DOI:** 10.1093/nsr/nwae176

**Published:** 2024-05-17

**Authors:** Jian Peng, Dongmei Xu, Zihan Xu, Hui Tang, Hong Jiang, Jianquan Dong, Yanxu Liu

**Affiliations:** Technology Innovation Center for Integrated Ecosystem Restoration and Sustainable Utilization, Ministry of Natural Resources, College of Urban and Environmental Sciences, Peking University, China; Technology Innovation Center for Integrated Ecosystem Restoration and Sustainable Utilization, Ministry of Natural Resources, College of Urban and Environmental Sciences, Peking University, China; School of Soil and Water Conservation, Beijing Forestry University, China; Key Laboratory for Environmental and Urban Sciences, School of Urban Planning and Design, Shenzhen Graduate School, Peking University, China; Technology Innovation Center for Integrated Ecosystem Restoration and Sustainable Utilization, Ministry of Natural Resources, College of Urban and Environmental Sciences, Peking University, China; School of Landscape Architecture, Beijing Forestry University, China; State Key Laboratory of Earth Surface Processes and Resource Ecology, Faculty of Geographical Science, Beijing Normal University, China

## Abstract

This study innovatively puts forward the three-stage restoration goals and cutting-edge key scientific issues of ecological restoration, as well as their relationships.

Ecological restoration is an effective measure to protect biodiversity, sustain ecosystem services and enhance social-ecological resilience. It is defined as the process of assisting the recovery of an ecosystem that has been degraded, damaged or destroyed [1,2]. Multiple large-scale ecological restoration initiatives have been launched by international agencies and governments in recent decades, such as the UN Decade on Ecosystem Restoration (2021–2030), the Convention on Biological Diversity, the New York Declaration on Forests, and the Sustainable Development Goals [1]. Extensive global ecological restoration priorities have made modest achievements; however they may not be efficient and lasting, and some of them have even failed, such as the Aichi Biodiversity Targets proposed by Global Biodiversity Outlook 5 (https://www.cbd.int/gbo/gbo5/publication/gbo-5-en.pdf). For example, some restoration projects planted trees in a disorganized fashion, and in an inappropriate place, manner and time, resulting in negative outcomes such as tree death and reduced ecosystem diversity. Multiple social-ecological elements and processes need to be incorporated into the ecological restoration of territorial space. Therefore, how do we develop more comprehensive and phased efforts to reverse ecosystem degradation trends and achieve ecological restoration goals? Here, we innovatively put forward and systematize the three-stage restoration goals and cutting-edge key scientific issues of ecological restoration, as well as their relationships.

The core goal of ecological restoration is the harmonious coexistence of humans and nature, which requires ecology as the foundation and culture as the vein. However, current restoration usually focuses on greening with low efficiency, and the separation dilemma of nature and society. In fact, there are three main goals of ecological restoration, corresponding to the three stages of ecological restoration (Fig. 1). The first-stage goal is regreening, which can be achieved by increasing leaf area index, vegetation productivity and vegetation coverage. The second-stage goal is beneficiating. Increasing efficiency ranges from enriching ecological functions to enhancing ecosystem services, and to strengthening human well-being. The third-stage goal is revitalizing, which refers to enhancing the physical resilience of ecosystems and the cultural resilience of social systems, ultimately achieving sustainability of ecological restoration. After clarifying the three-stage restoration goals, how to realize them can be tackled using the following 10 key scientific issues: reference state and size threshold before restoration, element coupling, trade-off efficiency, spatial connectivity, dynamic adaptation and scale cascade during restoration, and effectiveness evaluation, contribution distinguishment and cultural resilience after restoration. For each scientific issue, we systematically review the practical needs, existing or potential solutions, and research difficulties.

1. *Reference state*. How do we choose the reference state for ecological restoration? Clarifying whether the natural ecosystem has always been in the normal community succession historically, or has undergone ecological degradation, is critical to the feasibility of restoration, the assessment of restoration potential and the development of restoration planning. Reference states are regarded as undisturbed original ecosystems, top ecosystems with the same hydrothermal conditions or approximate ecosystems without ecological degradation integrating spatio-temporal perspectives [1,3]. While the historical states play the role of informing and referencing, enriching cultural connections, and revealing the future, they are not necessarily the most appropriate target for ecological restoration, and should be regarded as a guide rather than a template. Moreover, structure and function dimensions should be involved to evaluate ecosystems, so as to define whether ecosystems are similar in space. In general, considering ecological complexity and temporal change, reference states should focus on specific ecosystems that are realistic as restoration targets. However, an ecological restoration project may require multiple reference states, although the restoration effectiveness depends on the choice of reference states. Furthermore, high spatial heterogeneity, rapid environmental change and strong adaptive capacity may result in needing to adjust the reference state or adopt alternative states.
2. *Size threshold*. How much ecological land needs to be protected or restored to act as an ecological security barrier? Currently, some size thresholds of ecological land have been proposed, for example, 12% of the natural ecological carrying capacity should be reserved for biodiversity in ecological footprint accounting; no less than 25% of China's territorial area should be designated as the ecological protection red line; and 30% of land and sea should be protected for global biodiversity by 2030, and half of the Earth by 2050. However, the current setting of size thresholds mainly relies on restoration expectations, subjective willingness and financial capacity, while the dose-effect relationship, based on the resource and environmental carrying capacity, deserves more attention. Correspondingly, planetary boundaries and safe and just space were proposed as means of setting thresholds for critical planet biophysical processes [4]. It is also reported that the goals of minimum areas under specific conservation were determined based on subjective thresholds or supply–demand relationships, e.g. at least 44% of the terrestrial area requires conservation attention to safeguard global biodiversity [5]. Moreover, the quality and location of ecological space to be protected or restored, and the regulation of social-ecological elements or processes beyond specific thresholds, cannot be ignored, and should be further explored.
3. *Element coupling*. How should we embody the integrated protection and restoration of natural ecological elements such as mountains, rivers, forests, farmlands, lakes, grasslands, deserts and glaciers? How should we measure the interaction and coupling mechanisms among these ecological elements? Which social elements or processes contribute to or dominate the mechanisms? Unclear interaction pathways and associated processes have led to unknown coupling mechanisms and integrating approaches. Ideas of a social-ecological system, of nature-based solutions and metacoupling have provided possible solutions for element coupling. Ecological restoration has shifted from focusing on natural ecosystems to social-ecological systems, and from single-factor governance to multi-factor association [6,7]. A nature-based solution does not mean indulging ecosystem succession, but adopting a systematic approach, drawing on the development regularity of nature, focusing on the coupling association of all landscape elements and entire processes, as well as the whole region. Metacoupling was proposed to characterize human–nature interactions within and across adjacent and remote systems, including telecoupling, pericoupling and intracoupling [8]. Generally speaking, the next goal is to explore the role of specific elements as agents connecting all landscape elements, apart from the element of water which has been widely acknowledged. And it is also important to clarify the two-way feedback mechanism of human–Earth system coupling.
4. *Trade-off efficiency*. How do we avoid the social-ecological trade-off relationship under different restoration goals or objects? How should we enhance the trade-off efficiency between the cost and effectiveness of ecological restoration? How do we make the choice between artificial restoration and natural restoration, and further integrate them? Given limited resources, trade-offs are ubiquitous and unavoidable, such as between ecosystem services, biodiversity and ecosystem services, conservation costs and benefits, artificial and natural restorations, and different stakeholders. Taking biodiversity and food production as an example, agricultural diversification may reduce their trade-offs. It is indisputable that restoration strategies with long-term cost-effectiveness should be developed, rather than pursuing the maximization of a single metric or of short-term benefits. Approaches for the improvement of trade-off efficiency include the production possibility frontier and multicriteria optimization algorithms, which can identify the optimal combination of multiple products under the context of limited resources, maximize benefits and minimize costs [9,10]. Furthermore, comprehensive ecosystem monitoring and evaluation, and threshold identification of key social-ecological factors affecting resilience, are possible ways to determine whether artificial intervention, natural restoration, or their combination is appropriate. However, how to improve the social well-being equity of ecological restoration remains a great challenge, especially in view of the differentiated impact of ecological restoration on multiple stakeholders.
5. *Spatial connectivity*. Does the dispute over the ecological response of large patches and small patches of the same total area exist in ecological restoration? How can we enhance ecosystem structural and functional connectivity? Both large and small patches have important conservation value and play complementary roles, and their typical representative ecosystems are wilderness and urbanized areas, respectively. The expansion of protected areas provides an effective ecosystem-based spatial regulatory tool for enhancing ecosystem connectivity. The median area of newly established protected areas has decreased in recent decades [11], and there is a growing concern for smaller patches in global conservation. Rewilding is one of ecological restoration’s new approaches, aimed at reducing the human footprint and restoring ecosystem integrity [12]. The ecological network provides an effective means of enhancing ecosystem connectivity based on the habitat-corridor framework, which deepens the understanding of restoring complex systems and opens up new ways of measuring outcomes of ecological restoration [12]. However, how to integrate controlled experiments and scenario simulations in order to develop new indicators for characterizing spatial connectivity, and how to design ecological corridors for enhancing functional connectivity without other negative impacts such as species invasion and interference propagation, are still critical challenges that need to be addressed.
6. *Dynamic adaptation*. How should we dynamically adjust the phased goals of ecological restoration in the long-term restoration process of complex ecosystems under the context of adaptive management? How do we identify the thresholds for regime shift? Earth's complex systems often have alternative stable states, and when external disturbances break through the critical threshold, the shifts will occur between different stable states [13], so dynamic management is needed to enter a stable and controllable Earth system. Complex ecosystems require long-term restoration measures, and the time required for restoration increases with the complexity of phased goals. The phased goals of ecological restoration should be regulated by social-ecological feedback under the context of continuous monitoring. To maintain and optimize the stable equilibrium of the ecosystem, key variables of the social-ecological system need to be effectively regulated in order to avoid breaking ecological thresholds. Whether the energy inflow and outflow of the system are balanced, and whether the biogeochemical cycle is stable, can be used as criteria to determine whether the system has entered a steady state [13]. A couple of resilience-based indicators and approaches have been proposed to investigate regime shift. However, the time lag of restoration benefits should be further explored and considered in measuring ecosystem resilience.
7. *Scale cascade*. What are the target differences of ecological restoration carried out at different spatial scales? How do we choose the optimal scale of ecological restoration for different types of social-ecological systems? The spatial units of ecological restoration are mainly grids (with a spatial resolution of 30 m, 100 m, 1 km, etc.), administrative districts and watersheds, and different restoration objects should be focused on different spatial scales. Ecological restoration projects can achieve maximum net ecological benefits at appropriate spatial scales. Restoration targets at a larger scale (e.g. national carbon neutrality) cannot be key requirements for all restoration projects at smaller scales, although national restoration targets will finally be achieved by sub-national restoration projects. The size threshold issue is more significant at larger scales such as national or global scales, rather than smaller scales such as cities or counties. Multiple scales are hierarchical, interdependent and mutually constraining. In multi-scale systematic governance, there is a requirement to consider scale linkages, cross-scale extrapolation and integration of observations and analyses at different scales [6]. However, the multi-scale conduction methods of ecological restoration indicators and processes are still unclear, and the multi-scale coupling relationships of different social-ecological processes associated with ecological restoration need to be further explored.
8. *Effectiveness evaluation*. How should we evaluate the effectiveness of ecological restoration systematically and integrally? How should we determine the evaluation indicators and core criteria? Counterfactual scenario simulation and before-after control-intervention analysis are effective approaches to evaluating the effectiveness of ecological restoration, comparing the long-term trends of the evaluation indicators of restored and non-restored areas [14]. The most critical component is the selection of evaluation indicators characterizing ecological restoration effectiveness, together with the determination of indicator weights, which requires insights on ecosystem structure, ecosystem function, ecosystem service, biodiversity conservation, etc. The evaluation of restoration effectiveness focuses more on ecological outputs and neglects social-economic outcomes. The goal-cost-constraint framework can be adopted to systematically evaluate the efficiency of ecological restoration, and an indicator system can be established considering constraint conditions, so as to evaluate whether the objectives of ecological restoration are achieved with limited input or cost. However, the following key issues remain unclear: how should we balance comprehensiveness and specificity in determining evaluation indicators, especially considering the huge spatial differences in the social-ecological contexts of ecological restoration projects in China? What is the effectiveness difference between integrated and non-integrated ecological restoration (and how do we measure the difference)? How should we develop the effectiveness evaluation from quantification of ecosystem status to dynamic early warning in order to address the risk of future environmental change globally or locally?
9. *Contribution distinguishment*. How do we determine the natural and anthropogenic contributions to the outcomes of ecological restoration? How do we distinguish the contributions of multiple sub-projects in an integrated ecological restoration project? It is acknowledged that the phenomenon of vegetation greening is often caused by a combination of ecological and socio-economic factors. Natural restoration benefits from natural ecosystem succession, and climatic warming and humidifying, while artificial restoration depends on artificial measures by way of reconstructing ecosystems, such as planting trees and grasses, as well as reducing or even removing negative human interventions to natural ecosystems. Statistical approaches and control experiments or simulations are usually used to quantitatively distinguish between natural and anthropogenic contributions [15]. Moreover, it is important for the performance evaluation of ecological restoration projects to clarify the contribution of each specific sub-project, which can also be used for scheming horizontal ecological compensation between sub-project areas. One possible method is to identify the implementation time, implementation area and implementation object. In addition, the benefits brought by multiple contributing factors may not necessarily be linearly superposed, and the performance of specific ecosystem restoration countermeasures will vary in different regions. Hence, how to determine the contribution of different factors under nonlinear conditions, and what the dominant factors and associated mechanisms are for determining the differentiated performance of restoration countermeasures, remain critical questions.
10. *Cultural resilience*. What role do economic incentives and local culture play in short-term and long-term ecological restoration? How far can local knowledge be expanded? It has been verified in previous restoration practices that promoting the participation of local communities and associated stakeholders in ecosystem restoration activities can effectively reduce the risk of local restoration failure [1]. It is also well known that short-term economic and policy incentives cannot guarantee long-term sustainability of ecological restoration. Combining nature and culture to create an affinity between local residents and ecological restoration, which can break through the spatial and temporal boundaries of ecological engineering projects, provides a sustainable and cost-effective method, i.e. the cultural resilience approach. Potential conflicts and cooperation among different stakeholders should also be considered in ecological restoration planning. Compared with solely focusing on ecosystems, ecological restoration from the perspective of the social-ecological system will be more effective [7]. However, how to characterize and enhance cultural resilience, and what the differences and correlations are between cultural resilience and ecological resilience, are still unknown. In addition, although knowledge co-production is becoming more and more important in solving sustainability problems, beyond specific climate, vegetation, soil, or terrain, local culture is no longer active. Thus, as the core of cultural resilience, local knowledge diffusion and corresponding influencing factors need to be further explored.

**Figure 1.**
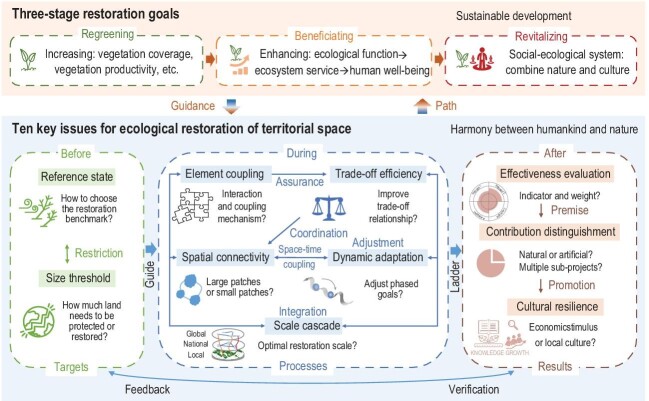
Three-stage goals and 10 key issues of ecological restoration.

There are also progressive and interrelated logical relations among these 10 key issues, which need to be considered in an integrated manner. In general, the goals set before restoration can guide restoration processes during restoration, which provide a ladder to restoration results after restoration. Restoration goals can be further used to verify restoration results, while the latter can be the feedback to the former. In the stage before restoration, the reference state and size threshold target are mutually restricted. During restoration, element coupling clarifies the element relationship, and trade-off efficiency refers to the coordination of element relationship. Then ecological restoration can be conducted through achieving both connectivity in the spatial dimension, and adaptation in the temporal dimension. Correlated with the other four issues, scale cascade is aimed at the integration of multi-scale ecological restorations. In the stage after restoration, both effectiveness evaluation and contribution distinguishment are the judgment of restoration performance, focusing on integrated evaluation indicators and the contribution of restoration sub-projects, respectively. Effectiveness evaluation is the premise of contribution distinguishment. Building cultural resilience is a low-cost measure to make ecological restoration effective in the long-term, and can also be regarded as a further enhancement of the anthropogenic restoration contribution, in order to achieve revitalization, the ultimate goal of three-stage restoration. Relatively speaking, few studies have been conducted on reference state, size threshold, element coupling, dynamic adaptation and cultural resilience, covering all the three stages of ecological restoration.

Despite many restoration efforts implemented worldwide, the global ecosystem continues to degrade. This may be partly due to the lack of scientific recognition and induction of key issues in the ecological restoration of terrestrial space. Careful consideration of the three-stage restoration goals and 10 key issues proposed in this study is conducive to the spatial allocation, temporal deployment and benefit enhancement of ambitious restoration initiatives, and will help them be organized and feasible globally and locally. It is also noteworthy that the proposed 10 key issues are not definitive, and it is hoped that they will trigger researchers, managers and stakeholders to seek a deeper understanding and more systematic discussion on the ecological restoration of territorial space. Furthermore, integrated, ecosystem-based and multi-stakeholder-involved techniques are indispensable to the expected outcomes of ecological restoration. Various transdisciplinary approaches, as well as transboundary cooperation, should be developed to address the multidimensional governance challenges of complex social-ecological systems. Integrating top-down and bottom-up participatory approaches can enhance the practicality and efficiency of large-scale ecological restoration projects. All in all, ecological restoration science, technology and policy need to collaborate more closely in order to enhance ecosystem diversity, stability and sustainability.
